# Glyceraldehyde-3-Phosphate Dehydrogenase: A Promising Target for Molecular Therapy in Hepatocellular Carcinoma

**DOI:** 10.18632/oncotarget.623

**Published:** 2012-09-07

**Authors:** Shanmugasundaram Ganapathy-Kanniappan, Rani Kunjithapatham, Jean-Francois Geschwind

**Affiliations:** ^1^ Department of Radiology & Radiological sciences, Johns Hopkins University School of Medicine, Baltimore, MD, USA

**Keywords:** 3-bromopyruvate, GAPDH, Glycolysis, HCC, Iodoacetate, Koningic acid, Methylglyoxal, Saframycin A

## Abstract

Hepatocellular carcinoma (HCC) is one of the most highly lethal malignancies ranking as the third leading-cause of cancer-related death worldwide. Although surgical resection and transplantation are effective curative therapies, very few patients qualify for such treatments due to the advanced stage of the disease at diagnosis. In this context, loco-regional therapies provide a viable therapeutic alternative with minimal systemic toxicity. However, as chemoresistance and tumor recurrence negatively impact the success of therapy resulting in poorer patient outcomes it is imperative to identify new molecular target(s) in cancer cells that could be effectively targeted by novel agents. Recent research has demonstrated that proliferation in HCC is associated with increased glucose metabolism. The glycolytic enzyme, glyceraldehyde-3-phosphate dehydrogenase (GAPDH), a multifunctional protein primarily recognized for its role in glucose metabolism, has already been shown to affect the proliferative potential of cancer cells. In human HCC, the increased expression of GAPDH is invariably associated with enhanced glycolytic capacity facilitating tumor progression. Though it is not yet known whether GAPDH up-regulation contributes to tumorigenesis *sensu stricto*, emerging evidence points to the existence of a link between GAPDH up-regulation and the promotion of survival mechanisms in cancer cells as well as chemoresistance. The involvement of GAPDH in several hepatocarcinogenic mechanisms (e.g. viral hepatitis, metabolic alterations) and its sensitivity to a new class of prospective anticancer agents prompted us to review the current understanding of the therapeutic potential of targeting GAPDH in HCC.

Glyceraldehyde-3-phosphate dehydrogenase (GAPDH) is a classical protein with enormous biochemical and biophysical interests owing to its functional significance in glucose metabolism. The focus on GAPDH has gained further momentum with recent discoveries unraveling its non-glycolytic (non-enzymatic) roles associated with cell death and diseases. Pharmacologically, GAPDH obeys the cardinal rules of “druggability” due to its disease relevance and the presence of an inhibitory site (e.g. catalytic domain of an enzyme, ligand-binding site of a receptor). Although, it has been estimated that at least 2-5% of the human genome (among ~30,000 genes) may form potential therapeutic targets [1], the key requirements of “druggability”, in the interests of pharmaceutical industry or any drug-development programs, restrict the number dramatically into fewer candidates. Nonetheless, the efficacy of the agent(s) targeting such a “druggable molecule” and the tolerable systemic toxicity and so on, will eventually determine the fate of successful clinical translation. Current advancement in understanding the cancer-related roles of GAPDH together with the sensitivity of tumor cells to its inhibition designates GAPDH as a potential molecular target in cancer treatment. Several elegant reviews by Sirover and others have delineated the functional diversity of GAPDH, particularly its participation in cell death and survival mechanisms [2-6]. The aim of the current review is to critically evaluate, from an interventional oncology perspective, the druggability of GAPDH and its therapeutic potential in liver cancer.

## Liver cancer and current therapeutic challenges

**Figure 1:**
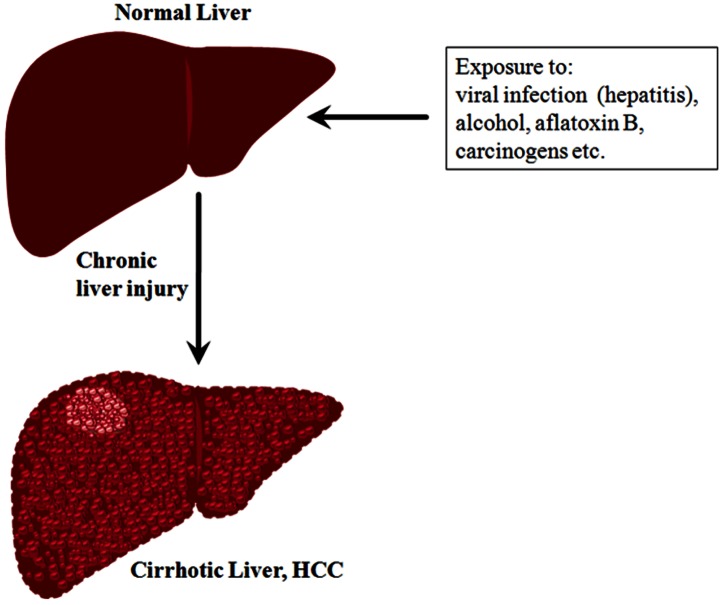
A schematic representation showing the transformation of normal liver into cirrhotic liver leading to HCC

Liver cancer is one of the most lethal malignancies and consistently ranked as the third most common cause of cancer-related death worldwide [7]. Intense research both at preclinical and clinical levels contributed a wealth of information on the causes and risk factors of liver cancer. In general, functional impairment of normal liver by hepatitis (due to the infection of hepatitis B and C viruses), excessive alcohol consumption, and aflatoxin B, in addition to oncogenic driver mutations, are considered as some of the causal factors that promote cirrhosis eventually leading to liver cancer [e.g. hepatocellular carcinoma (HCC)] (Figure 1). Statistically, the incidence of HCC in the Unites States is on the rise, secondary to a parallel rise in hepatitis C virus (HCV). In HCC, patient survival remains poor at less than nine months, and largely depends on the stage of the disease. Patients with intermediate-stage disease show better 3-year survival expectancy than advanced-stage disease (viz., 50% vs. 8%) [8]. Current curative therapies include surgical resection and transplantation, and are very effective in early-stage disease, however very few patients qualify for such treatments. The asymptomatic nature of HCC and the lack of an early-detection marker invariably result in the diagnosis of the disease at an advanced stage enforcing the patients to other treatment modalities. Among other therapeutic options for HCC, loco-regional therapies have the unique advantage of selective-targeting of tumors (primarily under image guidance), thereby evading systemic toxicity [9]. In clinics, the loco-regional therapies in practice consist of intra-arterial chemo-embolization or radio-embolization [10, 11] and percutaneous ablative therapies [12-15]. The effectiveness of loco-regional targeted-delivery in achieving tumor ablation greatly impacted the development of new, potential chemotherapeutic agents of high target-specificity and tolerable-toxicity profile. Nevertheless, as HCC is always associated with an underlying disease, it is imperative to administer any novel agent that selectively targets tumor cells with a high level molecular specificity. Recently, there has been a tremendous progress in the development of various therapeutics targeting different pathways or molecules of HCC[16, 17], yet the therapeutic success is often counteracted by chemoresistance and tumor recurrence necessitating the search for sensitive target molecule(s) that can be effectively targeted by potent inhibitors/drugs.

## Rationale for molecular targeting of tumor metabolism in liver cancer

Liver cancer has long been known to have increased-glucose metabolism, a prominent biochemical-signature of solid tumors. This tumor-specific metabolic phenotype plays pivotal roles in several biosynthetic processes facilitating uninterrupted growth. Further, recent data indicate that oncogenic driver mutations culminate in altered signal transduction pathways enabling tumor cells to reprogram their metabolic circuitry to adapt to the microenvironment. For example, it has been demonstrated that enhanced nutrient uptake is an effect of oncogenic *RAS* mutations [18]. Similarly, the tumor suppressor, p53, which has been known to be mutated in majority of tumors, has a role in the regulation of glucose metabolism [19]. Mounting evidence indicates that the tumor-specific shift in metabolism is vital for the uncontrolled proliferation and invasiveness of almost all solid tumors. Alternatively, this disparity in glucose metabolism between tumor cells and normal cells suggests a window of opportunity in treating cancer. This altered metabolism has been exploited by PET imaging in clinical diagnosis in the detection of malignant tumors using the glucose analog, fluorodeoxyglucose (FDG). Thus, tumor (glucose) metabolism has been recognized as critical for tumor growth, hence aptly described as “Cancer's Achilles’ Heel” [20] accentuating it as a potential therapeutic target [21, 22]. Hence, the ramifications of disrupting glucose metabolism could be envisaged to generate desirable anticancer effects.

Strong data provide the scientific rational for targeting glucose metabolism in treating HCC, or liver cancer in general. HCC has long been known to demonstrate regulation of glycolytic enzymes facilitating aerobic glycolysis [23]. It has also been demonstrated that in HCC, tumor proliferation is tightly correlated with glucose metabolism [24]. Indeed, both increased-glucose metabolism and proliferation share common regulatory pathways, making tumor metabolism a unique therapeutic target [25]. The therapeutic potential of targeting tumor metabolism triggered a renewed interest in studying glucose-metabolism. Consequently our understanding of the molecular regulation of tumor glycolysis has advanced significantly [26]. Many new investigational agents with potential inhibitory effects on glycolysis have been developed, and evaluated both *in vitro* and *in vivo* models. Despite promising results in preclinical tumor models [27], the majority of these agents have not been successfully translated into the clinic thus far, either due to the lack of efficacy in the clinical setting and/ or significant systemic toxicity. Thus, the need to identify a molecular target that is indispensable for cancer cell survival and developing an agent to effectively inhibit the molecule remains critical for successful anticancer therapy.

## GAPDH in hepatocarcinogenic mechanisms

Several reports unravel the participation of GAPDH in pathways that are cross-linked with cancer-specific or cancer-related phenotypes. GAPDH has been known to interact with the nucleic acids of Hepatitis B [28, 29] and C [30] viruses that cause hepatitis, a major contributing factor for hepatocarcinogenesis. Although GAPDH binding with nucleic acids of other viruses have also been reported (e.g. influenza virus, Japanese encephalitis virus) [31, 32], none of those viruses have been significantly associated with hepatocarcinogenesis or any other carcinogenesis.

Accumulating data indicate a strong link between GAPDH up-regulation and tumorigenic potential of transforming cells. For example, granulocyte macrophage (GM) colony-stimulating factor-1 (CSF-1), a factor known to play a pivotal role in several malignancies, has been increasingly recognized to be associated with hepatocarcinogenesis [33-35]. Surprisingly, GAPDH has been known to bind with CSF-1 mRNA resulting in increased stability, thus contributing for tumorigenic potential or malignant phenotype [36]. Moreover, analysis of HCC patient samples revealed the incidence of GAPDH up-regulation in human HCC strongly correlates with *c-jun*, a proto-oncogene that has long been known to be involved in liver tumorigenesis [37]. It remains to be seen if such a correlation exists between GAPDH expression and other oncogenes (e.g. *KRAS*) in HCC progression. Though it is not known whether GAPDH up-regulation contributes for tumorigenesis *sensu stricto*, adequate data unequivocally demonstrate the existence of an association between GAPDH over-expression and pro-survival mechanisms [5] and chemoresistance [38] in cancer cells.

**Figure 2:**
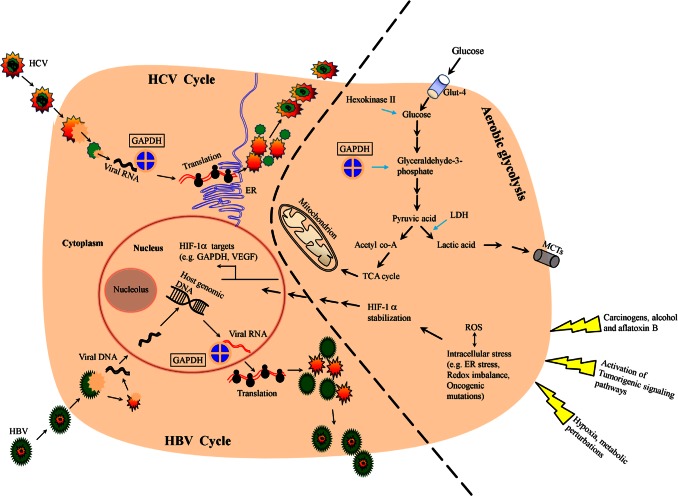
A schematic showing the involvement of GAPDH in hepatocarcinogenic mechanisms

Metabolically, in human HCC, the increased expression of GAPDH is invariably associated with increased glycolytic capacity [23, 39], facilitating tumor progression (Figure 2). Recently, a hitherto unknown role of GAPDH in the regulation of mammalian target of rapamycin (mTOR)-complex1 (mTOR-C1) signaling pathway has been documented [40]. mTOR pathway is a growth signaling mechanism that has been active during hepatocarcinogenesis [41, 42], hence provides an opportunity for therapeutic targeting [43-48]. It has been found that GAPDH regulates mTOR-C1 signaling according to the availability of glucose (hence ATP). GAPDH interaction with rheb, the activator of mTOR, mitigates mTOR-C1 signaling pathway thus halting the growth mechanism under conditions of energy depletion or inadequate glycolysis. It has to be noted that only chronic disruption but not transient inhibition of mTOR affects tumor cells [49]. Thus, GAPDH in addition to its role in glycolysis and energy production also signals and regulates other pathways like mTOR, depending upon cellular requirements. Hence, GAPDH is one of the very few earliest metabolic enzymes to exert such pleiotropic effects on energy metabolism and related signaling pathways. Unlike the majority of metabolic enzymes, GAPDH displays the unique feature of being functionally active in all three cellular compartments; nucleus, cytoplasm and plasma membrane. As a result, the interaction of GAPDH with either proteins or nucleic acids has been attributed to its diverse functions. Such interactions have been known to result in a variety of effects; (a) its interaction with VDAC-1 initiating pro-apoptotic membrane permeabilization of mitochondria [50], (b) its binding with tubulin affecting cytoskeletal structures and membrane trafficking and (c) its localization within endoplasmic reticulum (ER) affecting secretory pathway. GAPDH also interacts with RNA (e.g. human tRNA, lymphokine mRNA), and DNA (e.g. binding with telomere [38, 51]), and has been known to impact the genetic regulation of Oct-1 by participating in the OCA-s complex.

Such a multifaceted role for a single protein (GAPDH) would nevertheless require complex structural organization and regulation under dynamic physiological conditions. In this context the role of posttranslational modification (PTM) of GAPDH has been critically analyzed. It has been well documented that GAPDH undergoes one or more modifications such as acetylation, O-GlcNAcylation, S-nitrosylation, thiolation and “siah-1-binding” depending upon its cellular function. All of these PTMs have already been known to affect the enzymatic function of GAPDH [52]. In other words, PTMs that underlie GAPDH's non-glycolytic roles eventually affects its glycolytic function.

Since it has been established that GAPDH achieves its non-glycolytic functions through PTMs, compromising its glycolytic role conceivably, it would have serious consequences on the bioenergetics of cancer cells. Nevertheless, its cellular abundance and cancer-specific over-expression could compensate or satisfy for the variety of functions while maintaining tumor glycolysis. From an experimental therapeutic point of view, the multiple roles of GAPDH such as enzymic function (in energy metabolism), scaffold or adaptor protein (in vesicular trafficking) makes GAPDH an attractive molecular target to achieve desired anticancer effects. In other words, blockade of GAPDH would impair multiple pathways / mechanisms forcing cancer cells to become fragile, eventually causing their death.

## Targeting GAPDH to treat liver cancer

**Figure 3:**
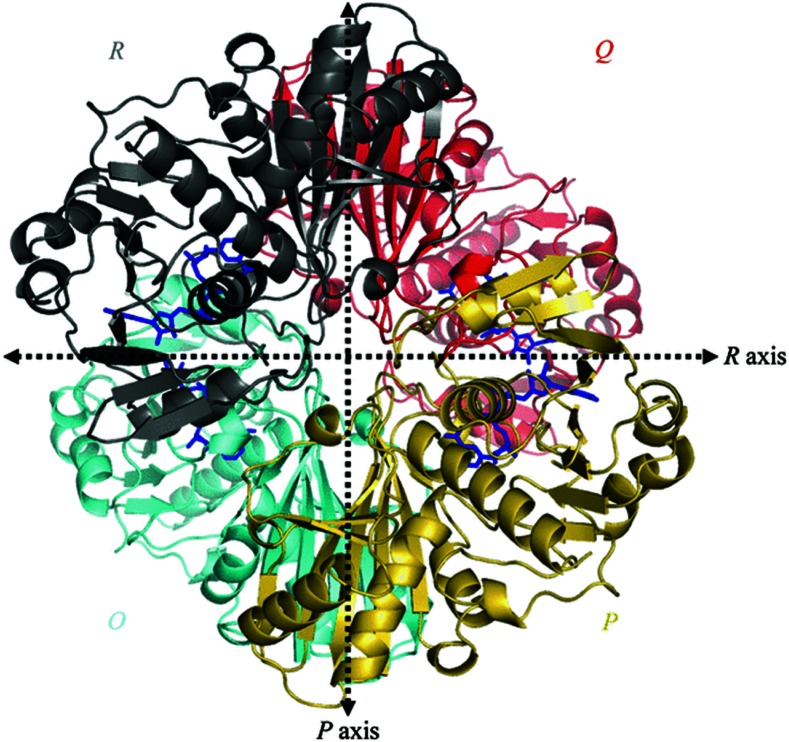
Overall view of the homotetramer of human liver GAPDH [44] (Reproduced with permission from http://dx.doi.org/10.1107/S0907444905026740)

Considering a therapeutic strategy to target GAPDH primarily relies on (a) its disease-relevance and (b) druggability. The rationale for considering GAPDH as a molecular target, from an oncologist's perspective, chiefly depends on its disease-relevance and the desirable phenotypic effects of abrogating its functions. Whereas, for a pharmacologist, the accessibility and inhibition of various domains of GAPDH by developing specific inhibitors to block its biochemical functions provides the rationale for drug design (Figure 3). GAPDH enzyme exists as a tetramer (four subunits) with each subunit having a catalytic site favoring the inhibition of the native enzyme even at any one of the four catalytic sites to disrupt its function.

Though enzymes such as lactate dehydrogenase (LDH) [53] and hexokinase II (HKII) [54] have been investigated in preclinical tumor models for their therapeutic potential, their respective inhibitors are yet to be successful in clinical trials. Hence, the need to identify a target that is critical for tumor growth but sensitive to therapy remains unanswered. As discussed elsewhere, the tumor specific roles of GAPDH include, apart from its glycolytic function, chemoresistance [38, 55], metastatic potential [56], protecting cells against caspase-independent apoptosis [5] and cell cycle regulation [57]. Thus, any interference with GAPDH's function is anticipated to surpass the effect of a single molecule (e.g. LDH, HK II) targeted-therapy that exclusively targets one molecule, and one pathway/function, where the interrupted physiology could be compensated by collateral or alternative mechanisms. Conceivably, GAPDH inhibition could sensitize the cancer cells for chemotherapy, as the protection and resistance offered by GAPDH will also be abrogated. However, the concern of ubiquitous nature of GAPDH, and the related systemic toxicity, needs to be addressed.

In this context, advancements in current interventional approaches (like loco-regional therapies, thermal-, cryo-, and chemo-ablative therapies, nanoparticle or lysosome-based drug delivery etc.) and identification of GAPDH-specific inhibitor(s) provide impetus in exploring the therapeutic potential associated with the anti-GAPDH therapeutic strategy. Thus, molecular targeting of GAPDH by tumor-specific delivery or inhibition could provide a viable therapeutic opportunity enabling us to overcome the current challenges in chemotherapy.

## Preclinical efficacy of GAPDH inhibitors

**Figure 4:**
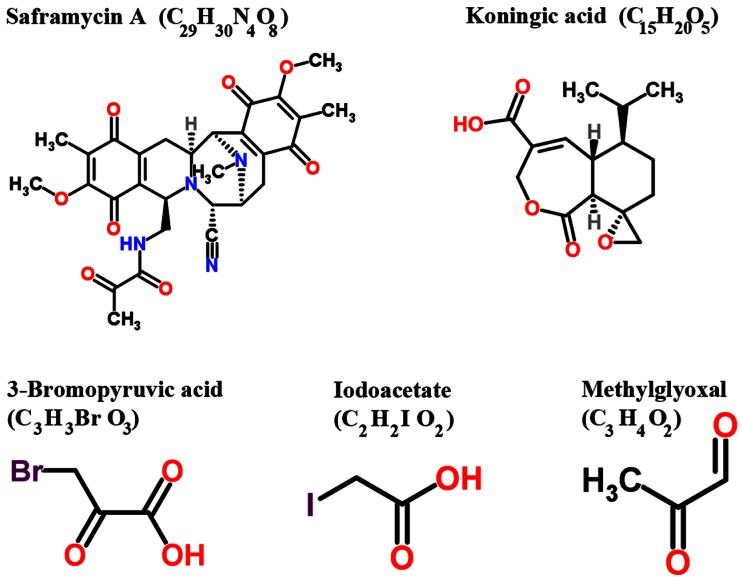
Structure of various inhibitors of GAPDH with anticancer effects in preclinical models (Reproduced with permission of RSC Worldwide Ltd from http://www.chemspider.com)

Several inhibitors derived from natural and / or synthetic sources have been investigated for their anti-GAPDH efficacy in treating cancer (Figure 4, Table I). Here, we discuss the agents that have been investigated in preclinical models that demonstrate adequate data on anticancer efficacy of such inhibitors.

**Table 1:** GAPDH antagonists in preclinical investigations

Antagonist	Key Reference(s)	Experimental Evidence
Iodoacetate	McKee et al. 1965	*In vitro* evidence for partial inhibition of GAPDH by iodoacetate
Koningic acid	Endo et al., 1985 Kumagai et al., 2008	*In vitro* evidence documenting the inhibition of GAPDH Ehrlich ascites suppression by GAPDH inhibition *in vivo*
3-Bromopyruvate (3-BrPA)	Barnard et al., 1993 Geschwind et al., 2002 Pereira de Silva et al., 2009 Ganapathy-Kanniappan et al., (2009) Ganapathy-Kanniappan et al., (2012)	First report indicating 3-BrPA binding with GAPDH First loco-regional therapy demonstrating anticancer efficacy of 3-BrPA First biochemical evidence in human HCC cells indicating that GAPDH and could be the primary target of 3-BrPA First autoradiographic evidence in human HCC cells demonstrating GAPDH as the primary target of 3-BrPA First report demonstrating percutaneous ablation of human HCC by targeting GAPDH through 3-BrPA
Methylglyoxal	Ray et al., 1997 Lee et al., 2005	Demonstrates GAPDH of tumor cells as the principal target *In vitro* evidence showing biochemical modification of GAPDH by methylglyoxal
Saframycin A	Xing et al., 2004	*In vitro* evidence demonstrating GAPDH as the target of Saframycin A –DNA adducts. Suggests GAPDH could be a chemotherapeutic target
Oligonucleotide siRNA shRNA	Kim et al., 1999 Phadke et al., (2009) Ganapathy-Kanniappan et al., (2012)	First report showing that GAPDH inhibition by antisense oligonucleotides affects proliferation, and induces apoptosis in cancer cells Demonstrates that GAPDH-siRNA induces cell cycle arrest First report demonstrating antitumorigenic effects of GAPDH silencing in human HCC both *in vitro* and *in vivo*

### Saframycin (Saf A)

Myers and his co-workers [58] elegantly demonstrated the involvement of GAPDH in the anti-proliferative effects of Saframycin A (Saf A), a natural product of bacterial fermentation. This report suggested GAPDH as a protein target of chemotherapeutic agents. However, the principal mechanism underlying the anti-proliferative effects of Saf A involves the formation of DNA adducts as the first step followed by the binding of the drug-DNA adduct with GAPDH protein. Although GAPDH is implicated in the anti-proliferative effects mediated by Saf A class agents, additional experimental evidence is required to demonstrate if Saf A is a specific inhibitor of GAPDH.

### Koningic acid (KA)

The fungal metabolite Koningic acid (KA) has been reported to inhibit glycolysis through the inhibition of GAPDH [59, 60]. The antiglycolytic principle of KA has been documented in multiple *in vitro* models. The efficacy of KA has been shown to be directly proportional to the glycolytic- dependency of cells, with cells exhibiting increased glycolysis demonstrating higher sensitivity [61]. KA binding site with GAPDH and the subsequent inhibition of activity has been identified. Preliminary reports have also demonstrated that administration of KA within 8 days of intraperitoneal inoculation of Ehrlich ascites tumor cells provided survival benefit to mice compared to untreated placebo, although detailed reports on the effect of KA on the rate of tumor growth and other tumor types are wanting. Further investigations on KA with a focus on selective targeting of tumor-GAPDH and not normal cellular GAPDH would provide an opportunity in understanding and advancing its therapeutic potential.

### 3-Bromopyruvate (3-BrPA)

The metabolic blocker, 3-bromopyruvate (3-BrPA), a halogenated analog of pyruvic acid, has gained significant attention due to its remarkable antitumor effects. *In vitro* testing against human HCC cells demonstrated that 3-BrPA inhibited glycolysis and blocked ATP production causing apoptosis in a dose-dependent manner [62]. Tracer studies with radio-(^14^C)-labeled 3-BrPA demonstrated GAPDH as the primary intracellular target of 3-BrPA. The binding of 3-BrPA to GAPDH in multiple cell lines [63, 64] substantiated the fact that GAPDH inhibition underlies 3-BrPA's antiglycolytic effect, leading to apoptotic cell death [64]. 3-BrPA is one of the very few agents that have substantial data demonstrating GAPDH as the preferred target. Unlike other alkylating agents, 3-BrPA demonstrated tremendous specificity in molecular targeting, enforcing its antitumorigenic effects by promoting energy depletion, disruption of redox balance and induction of intracellular stress in a concurrent fashion. Therefore it appears that 3-BrPA is an extremely promising agent due to its tumor selectivity and ability to promote a multipronged antitumor effect enabling it to progress towards clinical trials.

### Iodoacetate (IA)

McKee et al., [65] provided the earliest documentation of Iodoacetate (IA)'s inhibitory role on GAPDH in cancer cells, where the effect of GAPDH inhibition was seen in micromolar concentrations of IA. Interestingly at the lowest concentration (3 micromoles) used by these investigators the cancer cells switched from glycolysis to oxidative phosphorylation (as evident by an increase in oxygen consumption and a decrease in lactate production) with no signs of cytotoxicity. However, currently IA's use as a metabolic blocker is at higher concentrations (~100 micromoles) where significant cytotoxicity occurs [66]. Although the metabolic inhibitory effect of IA has been attributed to its effect on GAPDH activity, further investigations are required to validate as GAPDH is the primary target of IA.

### Methylglyoxal (MG)

Methylglyoxal (MG) a normal metabolite of glucose metabolism is formed during the process of glycolysis by dephosphorylation of glyceraldehyde-3-phosphate or dihydroxyacetone [67]. Methylglyoxal has been reported to inhibit tumorigenesis by interfering with the growth of malignant cells [68]. In the past decade reports have indicated that methylglyoxal targets the enzyme GAPDH, and this inhibition is the primary mechanism underlying its anticancer effects [69]. Lee et al., [67] have also demonstrated that the physical interaction / binding of methylglyoxal resulted in an alteration of the structure of GAPDH leading to inactivation. Methylglyoxal mediated glycation has been attributed as the cause for such an inactivation of GAPDH. Although very interesting, additional experimental evidence validating the tumor selectivity and molecular specificity (showing GAPDH as the only or at least the primary target) of MG in multiple tumor models will help further advancement of MG.

### RNAi

Several reports indicate that silencing GAPDH by antisense oligonucleotides [70] or small-interfering (si) RNA [57] induces apoptosis or affects cell proliferation, *in vitro*. To our knowledge, until our recent documentation [71], there was no report on the plausibility of shRNA-mediated GAPDH knockdown in the management of HCC. Yet, two major challenges to this approach remain. They are; (i) the ubiquitous nature of GAPDH, raising the concern for non-specific toxicity and (ii) its intracellular abundance, which would require higher doses of drug for therapeutic effect. To overcome these challenges, loco-regional therapies such as percutaneous and intra-arterial deliveries provide a viable alternative to systemic administration [72, 73]. The unique advantage of selectively targeting tumors under image guidance would minimize systemic toxicity [9]. Data demonstrated that percutaneous injection of GAPDH-antagonists block HCC progression. Future studies on the optimization of treatment regimen as well as delivery system with GAPDH antagonists will enable us to characterize the means of achieving maximal therapeutic efficacy either alone or in combination with other therapies. For instance, nanoparticle based slow release of potential therapeutic agents has emerged as a promising approach in preclinical models [74, 75]. Hence, integration of GAPDH antagonists with nanoparticles will allow us to improve the efficacy of therapy with minimal number of percutaneous injections, which is feasible in clinical translation. In summary, molecular targeting of GAPDH *via* percutaneous injection of either an inhibitor, 3-BrPA, or shRNA blocks tumor progression demonstrating the therapeutic potential of targeting GAPDH in HCC.

## Selective targeting of GAPDH

The involvement of GAPDH in several mechanisms that are associated with hepatocarcinogenesis (e.g. viral hepatitis, metabolic alterations), and the sensitivity of human HCC to targeted inhibition of GAPDH underscore the therapeutic potential of inactivating GAPDH, particularly in liver cancer. Next, the efficacy of targeted inhibition and the necessity of preventing systemic toxicity are the tasks to be achieved for clinical translation of such anti-GAPDH approach.

## Loco-regional therapies

Image-guided procedures, especially intra-arterial therapies, play a key role in the treatment of patients with liver cancer [10, 76]. The advantage of loco-regional approaches is that it provides not only access to the core but also to the periphery of the tumor. In addition, much greater drug concentrations can be achieved within tumors while minimizing systemic exposure. For example, the intra-arterial delivery of 3-BrPA in animal models of liver cancer demonstrated striking antitumor effects [77, 78] in most cases significantly prolonged the survival [71, 79]. In addition, combinatorial approaches can also be envisaged for improved or better effects in cases where such dual therapies are required. Recently, a clinical investigation revealed a marked effect of combining sorafenib with drug-eluting beads where the patient response was significant [80], assuring the feasibility and effectiveness of such loco-regional therapies.

## Tumor specific silencing of GAPDH

The therapeutic potential of RNA interference (RNAi) strategy for treating HCC has been increasingly recognized [81]. Despite attempts by several groups, the only RNAi therapeutic against liver tumor that has entered Phase I trial is ALN-VSP02 that targets kinesin-spindle protein and vascular endothelial growth factors (ClinicalTrials.Gov, identifier # NCT00882180). Thus the translation potential of RNAi mediated blockade of HCC is largely unexplored. Targeting tumor-specific GAPDH could be achieved either by targeted-delivery or selective-activation of si/shRNA in the tumor. The targeted delivery is within the limits of loco-regional therapies whereas selective activation involves a strategy of molecular cloning. For instance, if the expression of GAPDH-shRNA is regulated by the known marker of HCC (the alpha-fetoprotein, AFP) it would enable us to selectively target AFP-positive HCC. In such a scenario, even if the GAPDH-shRNA inadvertently enters into normal, non-cancerous cells, the toxicity will be prevented as the silencing effect is under the control of the presence of HCC marker, AFP.

## FUTURE DIRECTIONS

**Figure 5:**
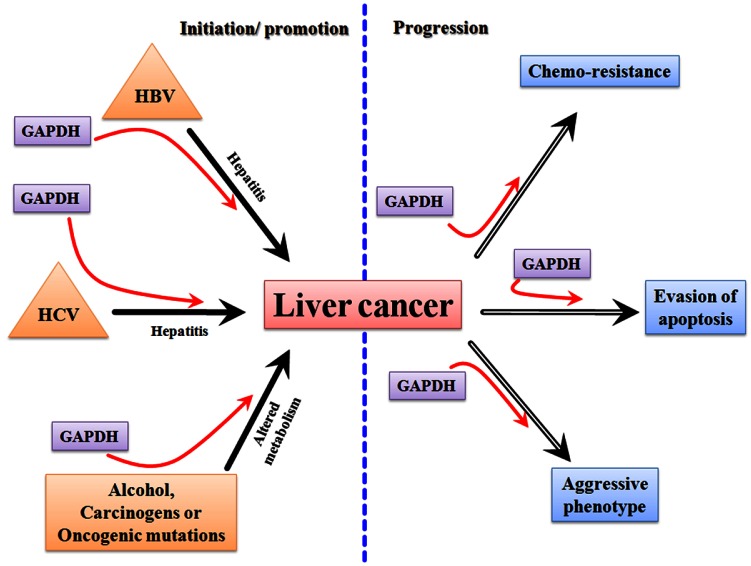
A schematic diagram showing the involvement of GAPDH in the processes related to the initiation/ promotion and progression of hepatocarcinogenesis

Successful translation of any potential chemotherapeutic for cancer essentially relies on the molecular-specificity of the therapeutic agent and its selectivity in targeting tumor. However, for liver cancer such as HCC, there is an additional requirement that needs to be fulfilled for successful practice in the clinics; that is the potential new drug candidates must exhibit an extremely favorable toxicity profile in order to preserve the functionally normal liver. This necessity is due to the fact that liver cancer typically arises in the context of an underlying liver disease (e.g. cirrhosis) making the liver especially susceptible to any kind of therapeutic insult. Thus ideal molecular targets will be those that are critically required for cancer cell proliferation, chemoresistance and metastasis. Since GAPDH is known to be involved in all of these processes (Figure 5) it is an extremely attractive target for therapy.

**Figure 6:**
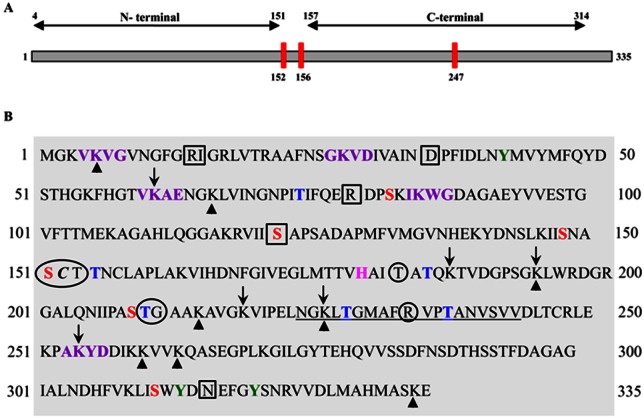
Human liver GAPDH showing various motifs (A). Line diagram showing the N-terminal and C-terminal regions with Cysteine residues (red bars). The amino acid residues numbered as 152 to 156 correspond to the catalytic site. Note: two cysteine residues located in the catalytic site are sensitive targets for majority of the GAPDH inhibitors. **(B).** The peptide sequence of GAPDH subunit indicating various posttranslational modification sites. Circles (○) represent Glyceraldehyde-3 phosphate binding sites; Square Boxes (□) represent NAD binding sites; Red font represents phosphoserine sites; Blue font represents phosphothreonine sites; Pink font represents aminoacid residue that Activates thiol group during catalysis; Green font represents phosphotyrosine sites; Purple font represents Predicted Sumoylation sites (70-90% probability); Italic font represents S-nitrosylation site and Underlined font represents SIAH-1 binding site. Arrow-head (↑) indicates predicted methylation sites while the with Down-ward Arrow (↓) indicates acetylation sites. Region 2-148 is the domain involved in the interaction WARS (tryptophan-tRNA ligase).

Identifying a specific inhibitor can be as difficult as identifying the molecular-target. Based on our current understanding of GAPDH and its functions, it is evident that inhibition of either the glycolytic role or interference with its non-glycolytic actions is sufficient to block GAPDH. Akin to this, several motifs have been predicted in the peptide sequence of GAPDH that can be attributed to its multiple functions (Figure 6). Preclinical evaluation of GAPDH inhibitors to date has been shown to inhibit its glycolytic role. Whether such inhibitors affect the PTMs, and their anticancer effects are PTM-inhibition dependent, have yet to be proven. It certainly appears that PTM remains an area that requires intense research especially since the majority of the non-glycolytic functions of GAPDH are affected by PTMs. For example it is known that phosphorylation of the Thr 237 decreases the nuclear translocation of GAPDH and blocks its apoptotic role [82]. Presumably, screening and developing inhibitors that would inhibit Thr 237 phosphorylation could restore GAPDH's-apoptotic role. Likewise, Akt2 kinase has been documented to mediate phosphorylation of Thr 237. Hence inhibitors specific for Akt2 kinase could support GAPDH dependent apoptosis. Interestingly, another PTM, the O-GlcNAcylation of GAPDH at Thr (227) has been shown to allow its nuclear translocation to facilitate its non-glycolytic functions [83] suggesting O-GlcNAcylation as a possible target. However, the translational potential of such an anti-GAPDH approach lies in the successful treatment of HCC in clinically relevant experimental models. Hence, further research is necessary in order to evaluate (a) the feasibility of delivery of GAPDH-shRNA under Ultra-sound guidance to target GAPDH in an orthotopic liver tumor model and (b) the molecular specificity of AFP-dependent silencing of GAPDH in HCC.

Recently, gene (silencing) therapy and/ or antibody based therapeutics have been shown to be very effective in loco-regional therapies however, their selectivity in targeting tumor cells and sparing normal hepatocytes would require tumor cell-specific functional activation of such therapeutics. This is plausible, if the functional activation of such therapeutics is dependent on the presence of a tumor-specific protein (e.g. AFP in some cases of HCC). Preclinical studies in line with this principle of tumor-specific functional activation have been documented and further studies are required to evaluate this selective silencing of GAPDH in HCC in relevant models mimicking the clinical set-up (e.g. spontaneous HCC, orthotopic liver tumor, image-guided therapy etc).

The failure of majority of the antimetabolites during clinical translation may be attributed to the plasticity of tumor cells where “alternative or complementary feeder pathways” can substitute for the disrupted metabolism. In other words, the complex network of tumor metabolism demonstrates innate flexibility to overcome or withstand disruption of any single biochemical pathway or inhibition of any particular molecular target. Hence, concurrent inhibition of complementary metabolic pathways (e.g., glycolysis, PPP, cellular redox balance) might prove to be a vital therapeutic strategy.

In summary, “*targeting early steps of glucose metabolism (e.g. GAPDH activity) will enable us to block intracellular energy production and other critical pathways linked to it (e.g. macromolecular biosynthesis), eventually causing cancer cell death”*. Future research focusing on the targeted (*via* loco-regional therapy) and selective (e.g. 3-BrPA) inhibition of GAPDH would provide us an opportunity to acquire better armamentarium in treating HCC.
